# Assessment of the level and distribution of health system responsiveness in Oyo State, Nigeria

**DOI:** 10.1186/s12913-022-08276-9

**Published:** 2022-07-12

**Authors:** Adeyemi Adelabu, Oluwaseun Akinyemi, Ayodeji Adebayo, Blessing Oladokun

**Affiliations:** 1 Nigeria Centre for Disease Control, Abuja, Nigeria; 2 Department of Health Policy and Management, College of Medicine, University of Ibadan, Ibadan, Nigeria; 3 Department of Community Medicine, College of Medicine, University of Ibadan, Ibadan, Nigeria; 4 Department of Mathematics and Statistics, East Tennessee State University, Tennessee, USA

**Keywords:** Distribution, Health system, Health systems responsiveness, Levels

## Abstract

**Background:**

Health systems need to be evaluated to ascertain if they are meeting their objectives. There is an increased interest in health system responsiveness (HSR) as a means to appraise health systems. This becomes vital as we put people at the centre of integrated health systems and put a premium on their rights and perspectives. Thus, this study assessed the levels, distribution and factors associated with HSR in Oyo State.

**Methods:**

The study was a cross-sectional study with 717 adults, who had used an out-patient health facility in the preceding 12 months, interviewed using a semi-structured, interviewer-administered questionnaire. HSR was measured on a multi-domain and multi-item (7 domains and 20 items) 5-point Likert scale that was developed by the WHO to measure HSR globally. Summary scores were computed for level, distribution and the most important domains of HSR. Determinants of poor HSR were determined using binomial logistic regression. The level of statistical significance was set at 5%.

**Results:**

The overall level of HSR was 47%. The highest-rated domains were confidentiality (72%), dignity (64%) and choice (60%), while the least rated were prompt attention (43%) and communication (52%). The overall distribution of HSR was 0.228 (range of 0 to 1) with the domains of prompt attention (0.595) and choice (0.506) being the most unequally distributed. The most important domains were communication, prompt attention and dignity. The least important domains were choice and confidentiality. The factors associated with poor HSR (overall) were no formal education, (OR = 2.81; 95% CI: 1.35–5.86), primary education as the highest level of education (OR = 2.19; 95% CI: 1.28–3.75), poor socioeconomic class (OR = 1.86; 95% CI: 1.23–2.80), using a government-owned facility (OR = 1.56; 95% CI: 1.11–2.19) and not using the usual health facility (OR = 1.69; 95% CI: 1.13–2.53).

**Conclusions:**

The overall level of HSR in Oyo State was low with the domains of prompt attention, communication and autonomy being the least rated domains. Therefore, concerted efforts should be targeted at improving HSR as this will improve wellbeing, health system utilization, and the overall health system.

**Supplementary Information:**

The online version contains supplementary material available at 10.1186/s12913-022-08276-9.

## Introduction

Health systems have to be evaluated to ascertain if they are meeting the goals, they set out to achieve, which include health, fair financing and responsiveness [1]. While health systems have predominantly been evaluated by health outcomes and other proxy measures such as accessibility [2], there is an increased cognizance and interest in health system responsiveness (HSR) as a means to appraise these systems as we pay more attention to the rights of patients and put people at the centre of these integrated systems [3, 4]. HSR remains a key objective of the health system that should be assessed because it is important in its own right, in that it improves overall wellbeing, relates to basic human rights [5], and because it improves other goals of the health system [5, 6]. HSR can be an indicator for assessing how well health systems respond to people’s non-clinical needs [5].

HSR evolved as part of the world health organization’s (WHO) broader conceptual framework on health systems in 2000 [7] and is defined as “the ability of the health system to meet the population’s legitimate expectations regarding their interaction with the health system, apart from the expectation for improvements in health or wealth” [6, 7]. It is conceptualized as a multidimensional concept as shown by the WHO framework on HSR with eight domains and they include; four interpersonal factors (autonomy, communication, confidentiality and dignity,) and four structural domains (access to social support networks, choice, prompt attention and quality of basic amenities) [8]. These legitimate expectations were defined in terms of international human rights norms and professional ethics [9] and the domains were selected based on criteria which include being amenable to self-reporting, comparability, comprehensiveness and validation [6, 10].

Over time, health systems globally have become impersonal and inhumane in the way it treats users [11–13]. Dissatisfaction with health workers in many countries focuses on their discourtesy and arrogance in their interaction with patients [11, 14, 15]. Also, waiting times in healthcare can be tedious and are the subject of criticism of health systems around the world [16, 17]. When health systems fail to respond to legitimate expectations of the populace they serve, it invariably means that the basic rights of people that use the health systems are violated and the tenets of moral principles of health care practice are not respected [9]. Consequently, individuals have failed to access and utilize formal health systems and health care services even when these services will improve their health and they can afford the care [18]. For instance, disrespect and abuse during labour and delivery are important issues that affect women’s decisions to deliver in health facilities [19]. Empirical data also suggest low utilization of primary healthcare services in Nigeria [20, 21]. This is due to several reasons, amongst which are HSR barriers [22]. Furthermore, when patients are not satisfied with the care they receive, they tend not to give relevant important information regarding their care or comply with their treatment regimen [23–25]. This is a crucial consideration for chronic infectious diseases that can become resistant like HIV, tuberculosis and chronic non-communicable diseases (NCD) like diabetes and hypertension [24].

It is also noteworthy that HSRs are particularly prone to inequalities, affecting the most underserved in society [27–30]. HSR inequalities, especially along social groups and health status can worsen the barriers to accessing health services and lead to poor health outcomes in those discriminated against [26, 31]. Therefore, health systems must aim to achieve equity as much as possible, in addition to the three goals of the health system [1]. It must be responsive to everybody equally no matter their status in the society, where they live or their health status [1]. Consequently, as with health outcomes, it is not enough for the health system to have a high level of responsiveness, but it should also be fairly distributed across the population. Fairness means that “the health system responds equally well to everyone, without discrimination or differences in how people are treated” [7]. It is therefore pertinent to not only evaluate the level but also the distribution of HSR in the community and the factors that affect this distribution.

Health system performance and outputs tend to differ from country to country, even in countries with similar economies and health expenditures [4, 31–35]. What is more, there are also differences in sub-national components of the health system and types of health services. Therefore, it becomes pertinent to assess Oyo State’s health system in terms of the legitimate needs of the population it serves. The evaluation will help policymakers and hospital managers to understand which aspects of HSR work well and which work less and what groups of the population experience low levels of HSR. It will also help to know what aspects of non-clinical care are most important to the population and how we can improve HSR and consequently improve health outcomes. Furthermore, HSR is a stewardship function of health systems and the goal of HSR can be improved at little or no cost [7]. Although there is evidence to suggest that HSR is an important dimension in evaluating health system quality, performance, and its importance to attaining universal health coverage [31, 36], very little has been published locally in this regard. This study hopes to fill the knowledge gap in this respect. Thus, the study was conducted to assess the levels, distribution, most important domains, and factors associated with HSR in Oyo State.

## Materials and methods

### Study design and setting

The study was a community-based, cross-sectional study that was conducted in Oyo State, Nigeria. The state is one of the 36 states located in the southwestern part of Nigeria. It covers 28,454 km of landmass with an estimated population of 7,840,900 (projected from the 2006 population of 5,580,894 using an annual growth rate of 2.6%) [37]. The state has three levels of healthcare: primary, secondary and tertiary level of care, with 735 public health facilities and 935 registered private health facilities.

### Sample size and Study population

A minimum sample size of 710 adults who were residents of selected LGAs and had used an out-patient health facility in the preceding 12 months in the state were included in the study. This was estimated using the formula calculating sample size for a single proportion. Out-patient care was defined as any health care use not including an overnight stay in a hospital or long-term care in a facility. Residents who resided in institutional settings were excluded from the study.

### Sampling

A multistage cluster design was deployed for this study. Using the sample frame of LGAs obtained from Oyo State Secretariat, two LGAs (one rural, and one urban LGA) were selected by simple random sampling. In the second stage, two wards were selected by simple random sampling from each of the selected LGAs. In the final stage, two enumeration areas (EA) were selected from the sample frame consisting of a list of EAs obtained from the LGAs, making a total of eight EAs. Furthermore, 95 households were randomly selected for the interview in each selected EA.

### Data collection

Data collection took place over five weeks between January and February 2020, conducted by the investigators and six RAs who were university graduates. The RAs were trained over two days so that they were familiar with the research process in terms of how to secure informed consent, objectives of the study, sampling procedure, data collection tools and plan for data collection and interview techniques. Each interview lasted for about 25 min. All interviews were conducted privately to ensure confidentiality and that respondents freely aired their true opinion.

### Study instrument

The short version of the WHO world health survey (WHS) semi-structured interviewer-administered questionnaires on HSR was adapted for the collection of data [31]. (see appendix 1 in Additional file 1) Some minor modifications were made to make it more applicable to the Nigerian context, for example, local names were added to make the vignette scenarios more relatable. In addition, questions about travel time and waiting times were added to the domain of prompt attention to improve its reliability. The tool has been substantially assessed for feasibility, validity, and reliability globally and in Nigeria [6, 38–40].

The questionnaire included information on sociodemographics, health, facility characteristics, ratings of HSR, vignettes on HSR and important domains of HSR. HSR was measured with multi-domain and multi-item (7 domains and 20 items) 5-point Likert scale that was developed by the WHO to measure HSR globally. In addition, there were two vignette questions per domain of HSR. The vignettes described the experiences of hypothetical individuals within each of the domains [41]. They were used to ascertain respondents’ expectations of the health system and then adjust respondents’ subjective ratings of their health care for their expectations, thereby reducing the systematic bias due to differential reporting and maintaining heterogeneity [41]. The vignettes were originally developed by the WHO and used in the WHS [42, 43]. The survey questionnaires were pretested in Osun State, to test for clarity of questions, gain preliminary insight into their construct validity and refine any ambiguity. The questionnaire was translated to the most predominant language, Yoruba and back-translated to English to ensure that its original meaning was retained thereby ensuring the retention of validity.

### Data management and variables

Data were collected electronically using the kobo collect tool kit on android phones and analyzed on SPSS version 25. Data collected were checked daily for errors, completeness and appropriate corrections were made. Variables were recoded where appropriate. Records of respondents were regarded as missing if scores on HSR domains were missing and were excluded from the analysis.

The independent variables include individual characteristics (sociodemographics, health status) and health facility characteristics.

Wealth index (WI) was created using principal component analysis based on the information on physical assets owned by the household. Physical assets listed in the questionnaire included; videocassette recorder, stereo system, video camera, washing machine, vacuum cleaner, refrigerator, telephone line, mobile, computer, internet, magazine or newspaper subscription, security system for home, household help employed and another house [44]. It was analysed as a categorical variable indicating the quintiles where 1 represents respondents in the lowest income quintile and 5 those in the highest income quintile. Principal components analysis (PCA) was used to generate a weight for each item covered by the questions [44].

The principal component score was calculated using the formula [44]

$$\mathrm{PCn}\;=\;{\mathrm a}_{\mathrm m1}{\mathrm X}_1\;+\;{\mathrm a}_{\mathrm m2}{\mathrm X}_2\;+\dots\dots\dots\dots{\mathrm a}_{\mathrm{mn}}{\mathrm X}_{\mathrm n}$$

Where PC is principal component score, a represents the weight for the mth principal component and the nth variable and X is the variable. A WI score was calculated for each individual by weighting the response concerning each item about that household by the coefficient of the first principal component and summing the outcomes. This was used to generate quintiles classified into the lowest, second, middle, fourth and highest socioeconomic groups. This was later categorised into three groups namely, poor = 1, middle = 2 and rich = 3.

The main dependent variable was HSR, which is a multidimensional concept. Seven out of eight domains were considered for patients who had utilized outpatient units of health facilities in the past twelve months. The domains consist of autonomy (involved in decisions), choice (of health care provider), clarity of communication (of health care personnel), confidentiality (e.g. talk privately), dignity (respectful treatment and communication), prompt attention (e.g. waiting times), and quality of basic facilities.

### Statistical analyses

#### Score computation

Ambulatory score for HSR: A summary score was computed for HSR, the simple average of the score using a five-point scale (after adjusting for expectations) on all the relevant domains. HSR was evaluated on seven domains. Respondents were asked to rate their experience in each domain on a five-point rating scale ranging from 1 “very bad”, 2 “bad”, 3 “moderate”, 4 “good” and 5 “very good”, with each domain measured as a categorical variable for which there is an assumed underlying latent scale.

Vignettes were used to adjust for reporting heterogeneity [42]. The scale cut-off point for the HSR vignettes was calculated using a non-parametric technique described by King et al. [45] and Tandon et al. [46]. The formula is described below. The technique involves making numeric adjustments to the individual raw responsiveness scores [43]. This technique was chosen because it is straightforward, uncomplicated and requires no additional presumptions. A similar method was used by Geldsetzer et al. [47] and Li et al. [48].

HSR: Non-parametric approach (numeric adjustments to the raw responsiveness scores) [43]

$$y_i=\left\{\begin{array}{cc}1,&if\ast y_i<\mu_1\\2,&if\ast y_i=\mu_1\\3,&\;\;\;\;\;\;\;if\mu_1<\ast y_i<\mu_2\\4,&if\ast y_i=\mu_2\\5,&if\ast y_i>\mu_2\end{array}\right.$$

**…** given ${\mu }_{1} \& {\mu }_{2}$ are the minimum and maximum observed values from 2 sets of vignettes for each domain

… $*{y}_{i}$is the estimated responsiveness score before the adjustment; an average score obtained from each respondent (ranging from 1 to 5)

**…**${y}_{i}$is the adjusted responsiveness score for each respondent; the adjusted responsiveness score is bounded by a range of 1 to 2*k* + 1 (where *k* is the set of vignettes used for the study; 2 sets of vignettes were used per domain).

Following adjustment of the responsiveness score, the scores were rescaled to 0 to 100 for ease of interpretation as the number of question items per domain differs across domains, it was done as follows:

$$\widehat{y_i}=\frac{y_i}{2k+1}\ast100=\frac{y_i}{2\left(2\right)+1}\ast100=\frac{y_i}5\ast100;$$

$\widehat{{y}_{i}}$is the rescaled adjusted responsiveness score,

To obtain the total responsiveness score, the average of the rescaled adjusted responsiveness score from all seven domains was taken: $\widehat{{y}_{total }}= \frac{1}{7} (\widehat{{y}_{prompt} }+ \widehat{{y}_{dignity} }+ \widehat{{y}_{communication} }+ \widehat{{y}_{autonomy } }+ \widehat{{y}_{confidentiality} }+ \widehat{{y}_{choice} }+ \widehat{{y}_{quality } })$ [6].

*Where* $\widehat{{y}_{total }}$ *is the ambulatory score of HSR.*

This average is referred to as the overall ambulatory HSR score (see appendix 2 in Additional file 1) [6]. In addition, the unadjusted scores are also added to the additional file (see appendix 3 in Additional file 1).

The use of vignettes to adjust responses for participants’ expectations relied on two assumptions: response consistency and vignette equivalence [45, 47]. Response consistency was achieved by ensuring respondents had the same expectations for the hypothetical vignette patients as for themselves. Vignette equivalence was achieved by asking questions that all respondents interpreted in the same manner.

Level of HSR: This was obtained from the proportion of respondents whose average score was good or very good. The ambulatory HSR score gotten above was dichotomized into poor (score of ≤ 60) and good (score of > 60). The cut-off was chosen based on the fact that the questions were answered on a five-point Likert scale, with < 60 representing moderate to poor and > 60% representing good and very good. In addition, it was based on what was observed in literature to ensure comparisons [31, 49]. Dichotomization helped to avoid the bias that could have occurred due to the contraction of reporting scale. It also helped to avoid violating the assumptions of the regression model [50]. The average percentage of respondents reporting that their last interaction with the health system was good across the relevant domains is referred to as the overall level of HSR [6].

Distribution of HSR: Distribution of HSR in this study was measured in two ways. Firstly, respondents were asked if they were discriminated against for any reason while using the health system. In addition, an inequality index was calculated using individual mean difference described below [29, 51]. Individual-mean difference measures the differences between individual levels and the mean level observed in that population and is normalized to an index between 0 and 1, with 0 being perfect equality and 1 the most unequally distributed.

Estimation of the individual-mean difference (IMD) for inequality index was calculated using: [29, 51]

$${IMD}_{(\alpha ,\beta )}= \frac{\sum _{i=1}^{m}| {Y}_{i}-\mu {\left.\right|}^{\alpha }}{n {\mu }^{\beta }}$$

…where $$\alpha =\beta =1$$

Where *IMD is the individual mean difference*

Y_i_ is the responsiveness level for individual i

${\upmu }$ is the average level of responsiveness in the population

$\text{n}$ is the number of people in the population

The β coefficient determines the extent to which the inequality measures are relative to the mean or absolute. If α = β = 1; the measure is strictly relative to the mean.

Most important domains of HSR: Scores were allocated to how important respondents rated the domains of responsiveness. The domains were ranked according to their importance. A maximum score of 7 was assigned to the most important domain and a minimum value of 1 for the least important domain. The scores were then aggregated for each domain and scaled over 10 for ease of interpretation.

#### Data analysis

Summary statistics were generated and presented in frequency tables. Means and standard deviations were used for quantitative continuous variables, while proportions were used for qualitative variables. The outcome was reverse coded to examine the factors associated with poor overall HSR and its various domains. Binomial logistic regression was used to analyse the effect of the multiple independent variables listed above. The significance level for entering a variable into the model was set at 10% from the bivariate analysis. In addition, age [40, 49, 52], gender [38, 40, 53, 54], socioeconomic status [27, 40, 47, 54, 55], level of education [40, 52, 56] and place of residence [27, 47, 49, 57] were variables found to be important from literature and were also added to the regression model regardless of if they met the 10% level of significance. Furthermore, a test of co-linearity was conducted using the variance inflation factor to determine if the variables in the model are correlated with one another and the highest value obtained was 1.127 indicating that correlation was minimal. Odds ratios were calculated between independent and dependent variables. The significance level for all statistical tests was set at 5%.

### Ethical considerations

Ethical approval for this study was gotten from the Oyo State Ministry of Health Ethical Committee - Reference no 13/479/1067. In addition, permission was obtained from community heads to enter communities and informed consent was sort from respondents.

## Results

In all, a total of 760 respondents met the inclusion criteria, used health facilities in the preceding twelve months, and 717 completed the interviews giving a response rate was 94.3%. The background characteristics of the study participants are shown in Table 1.

**Table 1:** Respondents’ sociodemographic, self-rated health and health care characteristics (*N* = 717)

Characteristics	N	(%)
**Age group (years)**		
18–24	110	(15.3)
25–39	342	(47.7)
40–59	196	(27.3
≥ 60	69	(9.6)
**Mean Age**	$36.9\pm 13.7$	
**Gender**		
Male	205	(28.6)
Female	512	(71.4)
**Marital status**		
Single	120	(16.8)
Cohabiting/Married	561	(78.2)
Formerly married	36	(5.0)
**Highest level of education**		
No-formal	46	(6.4)
Primary	92	(12.8)
Secondary	370	(51.6)
Tertiary	209	(29.1)
**Place of residence**		
Urban	360	(50.3)
Rural	357	(49.7)
**Religion** ^†^		
Christianity	462	(64.4)
Islam	252	(35.2)
Traditional/Others	3	(0.4)
**Ethnicity**		
Yoruba	676	(94.3)
Others (Igbo, Hausa,.)	41	(5.7)
**Wealth Index**		
Poor	208	(29.0)
Middle	271	(37.8)
Rich	238	(33.2)
**Occupation**		
Professional/Managerial	78	(10.9)
Skilled & Partially skilled	226	(31.5)
Unskilled	341	(47.6)
Unemployed	72	(10.0)
**Health status**		
Poor	50	(7.0)
Good	667	(93.0)
**Disability status**		
Yes	38	(5.3)
No	679	(94.7)
**Chronic illness**		
Yes	41	(5.7)
No	676	(94.3)
**Last visit to health facility**		
Last 30 days	215	(30.0)
Last 3 months	235	(32.8)
Last 6 months	116	(16.2)
6–12 months	151	(21.0)
**Person in need of healthcare**		
Respondent	521	(72.6)
Respondents’ child	196	(27.4)
**Type of facility visited**		
Government hospital	476	(66.4)
Private hospital/NGO	241	(33.6)
**Reason for last visit**		
Communicable disease	438	(61.0)
Non-communicable disease	93	(13.0)
Preventive services	126	(17.6)
Others	60	(8.4)
**Level of facility visited**		
Primary	327	(45.6)
Secondary	349	(48.7)
Tertiary	41	(5.7)
**Facility visited was a usual place**		
Yes	577	(80.5)
No	140	(19.5)
**Expenses at Last Visit to Health Facility (₦)**	(n = 650)	(n = 650)
Below 1,000	139	(21.4)
1,000–4,999	311	(47.8)
5,000–9,999	107	(16.5)
10,000 or more	93	(14.3)
Average expenses (₦) Mean ± SD	6997.5 ± 720.7	6997.5 ± 720.7
**Covered by Health Insurance**		
Yes	56	(7.8)
No	661	(92.2)

The average age of respondents was 36.9 ± 13.7 years and there were more females 514 (71.7%). Most, 373 (52.0%) had secondary as the highest level of education and 269 (37.5%) were in the middle class of the WI. Majority, 674 (94.0%), of the respondents said they had good health. Two hundred and fifteen (30.0%) of the respondents visited the hospital in the last 30 days, while 235 (32.8%) visited in 3 months. Most of the respondents, 521 (72.6%), accessed care for their self and 438 (61.0%) sought care for communicable diseases. Four hundred and seventy-eight (66.7%) utilized government-owned hospitals and 349 (48.7%) used secondary level facilities. Respondents spent an average of **₦**6,997 on their health care.

**Table 2:** Levels of health system responsiveness by domains (*N* = 717)

Domains of HSR		N		(%)	
**Autonomy**					
Poor		326		(45.5)	
Good		391		(54.5)	
**Choice**					
Poor		289		(40.3)	
Good		428		(59.7)	
**Communication**					
Poor		342		(47.7)	
Good		375		(52.3)	
**Confidentiality of Information**					
Poor		201		(28.0)	
Good		516		(72.0)	
**Dignity**					
Poor		261		(36.4)	
Good		456		(63.6)	
**Prompt Attention**					
Poor		409		(57.0)	
Good		308		(43.0)	
**Quality of Environment**					
Poor		304		(42.4)	
Good		413		(57.6)	
**Overall Responsiveness**					
Poor		379		(52.9)	
Good		338		(47.1)	

Table 2 shows the level of HSR for the different domains. In total the overall level of HSR in Oyo state was 47.1% with 338 respondents rating their experience of HSR as good. Confidentiality of information was the best-rated domain with 72% of respondents rating it as good, followed by dignity with 63.6%, and choice with 59.7%. The least rated domains were prompt attention and communication with 43.0% and 52.3% of respondents rating it as good respectively.

**Table 3:** Experience of discrimination by the health system

Reasons for discrimination	N	(%)
**Treated worse for any reason (*N* = 717)**		
** Yes**	21	3.1
** No**	695	96.9
**Treated worse due to Lack of Money (*n* = 21)**		
** Yes**	11	52.4
** No**	10	47.6
**Treated worse due to Social Class (*n* = 21)**		
** Yes**	7	33.3
** No**	14	66.7
**Treated worse due to Type of Illness (*n* = 21)**		
** Yes**	6	28.6
** No**	15	71.4

Table 3 shows the proportion of respondents that experienced discrimination while using the health system. When asked the direct question on discrimination, a total of 22 (3.1%) respondents reported they had experienced some form of discrimination in their interaction with the health system. The reasons cited for this discrimination were lack of money, social class, and type of illness.

**Table 4:** Distribution of health system responsiveness in Oyo State

Domains of HSR	HSR inequality index
**Autonomy**	0.483
**Choice**	0.506
**Communication**	0.497
**Confidentiality of Information**	0.409
**Dignity**	0.452
**Prompt Attention**	0.595
**Quality of Environment**	0.453
**Overall Responsiveness**	0.228

Table 4 shows the distribution of HSR in Oyo State using the inequality index which was measured by the differences between individual levels and the population mean level observed. The inequality index for overall HSR was 0.228 (range of 0 to 1) with 0 indicating perfect equality and 1 indicating the most inequalities. Accordingly, the domains that showed the most inequalities were prompt attention (0.595), choice (0.506) and communication (0.497). Confidentiality (0.409) dignity (0.452) and quality of amenities (0.453) showed the least inequalities among the domains.

**Table 5:** Dimensions of health system responsiveness that are most important to people

Domain	Total
	**Mean and SD**
**Autonomy**	$5.6\pm 3.2$
**Confidentiality**	$5.5\pm 2.9$
**Choice**	$4.4\pm 3.1$
**Communication**	$8.1\pm 2.0$
**Dignity**	$6.9\pm 2.8$
**Prompt Attention**	$7.9\pm 2.2$
**Quality of Amenities**	$6.1\pm 2.6$

Table 5 shows the most important domains of HSR. Overall, communication was rated as the most important domain, followed by prompt attention, dignity and quality of basic amenities. Choice was rated as the least important domain.

The results for factors associated with poor HSR are presented in Table 6. In the autonomy domain, those in the poor WI had a significantly higher odds of experiencing poor autonomy (OR = 1.78; 95% CI: 1.20–2.66). For the communication domain, respondents with no formal education (OR = 3.62; 95% CI: 1.73–7.57) and those with primary as the highest level of education (OR = 1.98; 95% CI: 1.18–3.33) had a significantly higher odds of experiencing poor communication. There were no significant factors associated with poor confidentiality in the logistic regression. Poor socioeconomic class (OR = 1.93; 95% CI: 1.28–2.92) and middle socioeconomic class (OR = 1.61; 95% CI: 1.09–2.37), using a private health facility (OR = 1.44; 95% CI: 1.02–2.02) and urban residence (OR = 1.45; 95% CI: 1.04 − 2.01) were significantly associated with experiencing poor choice.

**Table 6:** Logistic regression showing factors associated with poor health system responsiveness

**Variable**	**Autonomy**	**Communication**	**Confidentiality**	**Choice**
	**OR (95%CI)**	**OR (95%CI)**	**OR (95%CI)**	**OR (95%CI)**
**Age**				
$\ge 60$ years	Ref	Ref	Ref	Ref
40–59 years	1.11 (0.63–1.95)	0.60 (0.32–1.04)	1.03 (0.54–1.97)	0.76 (0.42–1.36)
25–39 years	1.10 (0.64–1.88)	0.92 (0.53–1.58)	1.09 (0.59–2.00)	0.87 (0.50–1.51)
18–24 years	0.99 (0.53–1.87)	1.02 (0.54–1.94)	1.52 (0.76–3.07)	1.30 (0.68–2.48)
**Gender**				
Female	Ref	Ref	Ref	Ref
Male	0.89 (0.63–1.25)	0.95 (0.68–1.34)	1.17 (0.80–1.70)	1.13 (0.80–1.61)
**Marital status**				
Married/cohabiting	NA	Ref	Ref	NA
Single/Formally married		1.17 (0.79–1.75)	1.40 (092–2.12)	
**Education**				
Tertiary	Ref	Ref	Ref	Ref
Secondary	0.96 (0.69–1.37)	1.04 (0.73–1.50)	1.36 (0.90– 2.04)	0.89 (0.61–1.29)
Primary	1.51 (0.90–2.50)	1.98 (1.18– 3.33)*	1.18 (0.66–2.11)	1.39 (0.83– 2.34)
Non-formal	1.65 (0.84–3.22)	3.62 (1.73–7.57)*	0.93 (0.43–2.01)	1.46 (0.74–2.87)
**Wealth Index**				
Rich	Ref	Ref	Ref	Ref
Middle	1.32 (0.91–1.92)	1.11 (0.77–1.61)	1.21 (0.80– 1.84)	1.61 (1.09–2.37)*
Poor	1.78 (1.20–2.66)*	1.37 (0.92–2.05)	1.29 (0.83–2.00)	1.93 (1.28–2.92)*
**Ownership of Facility**				
Private	Ref	Ref	Ref	Ref
Government	1.31 (0.95–1.81)	1.15 (0.83–1.60)	1.08 (0.75–1.56)	1.44 (1.02–2.02)*
**Place of residence**				
Urban	Ref	Ref	Ref	Ref
Rural	0.95 (0.67–1.24)	1.09 (0.79–1.51)	0.80 (0.56–1.13)	1.45 (1.04 − 2.01)*
**Self-rated health status**				
Good health	NA	NA	NA	NA
Poor health				
**Level of health facility**				
Tertiary facility	NA	Ref	Ref	Ref
Secondary facility		1.34 (0.67–2.72)	0.90(0.43–1.88)	1.35 (0.66–2.74)
Primary facility		1.04 (0.52–2.09)	0.92 (0.44–1.93)	0.99 (0.48–2.02)
**Usual place of care**				
Yes	NA	NA	NA	NA
No				
** Variable**	**Dignity**	**Prompt Attention**	**Quality of amenities**	**Overall responsiveness**
	**OR (95%CI)**	**OR (95%CI)**	**OR (95%CI)**	**OR (95%CI)**
**Age**				
$\ge 60$years	Ref	Ref	Ref	Ref
40–59 years	1.13 (0.62–2.05)	0.91 (0.52–1.60)	1.45 (0.80–2.65)	0.69 (0.38–1.25)
25–39 years	1.03 (0.59–1.82)	1.20 (0.70–2.06)	1.47 (0.80–2.60)	1.03 (0.59–1.80)
18–24 years	1.90 (0.99–3.66)	1.42 (0.75–2.69)	1.25 (0.63–2.40)	1.43 (0.73–2.80)
**Gender**				
Female	Ref	Ref	Ref	Ref
Male	0.87 (0.61–1.25)	1.03 (0.73–1.44)	1.04 (0.74–1.48)	0.87 (0.61–1.23)
**Marital status**				
Married/cohabiting	NA	NA	NA	Ref
Single/formerly married				1.16 (0.77–1.75)
**Education**				
Tertiary	Ref	Ref	Ref	Ref
Secondary	1.14 (0.77–1.68)	1.02 (0.71–1.46)	0.59 (0.29– 1.20)	1.22 (0.84–1.75)
Primary	1.41 (0.83– 2.41)	1.22 (0.73–2.05)	0.97 (0.57–1.64)	2.19 (1.28–3.75) *
Non-formal	2.13 (1.08–4.21)*	1.23 (0.62–2.42)	1.02 (0.71– 1.49)	2.81 (1.35–5.86) *
**Wealth Index**				
Rich	Ref	Ref	Ref	Ref
Middle	1.47 (0.98–2.18)	0.97 (0.67–1.41)	1.51 (1.03–2.23)*	1.36 (0.94–1.99)
Poor	1.67 (1.10–2.54)*	1.51 (1.01–2.25)*	1.53 (1.02– 2.31)*	1.86 (1.23–2.80)*
**Ownership of Facility**				
Private	Ref	Ref	Ref	Ref
Government	1.75 (1.23–2.49)*	1.46 (1.06–2.02)*	2.16 (1.54–3.04)*	1.56 (1.11–2.19)*
**Place of residence**				
Urban	Ref	Ref	Ref	Ref
Rural	0.76 (0.55–1.04)	0.76 (0.56–1.03)	1.84 (1.35–2.52)*	1.15 (0.83–1.59)
**Self-rated health status**				
Good health	NA	NA	NA	Ref
Poor health				1.03 (0.56–1.91)
**Level of health facility**				
Tertiary facility	NA	NA	NA	Ref
Secondary facility				1.14 (0.56–2.31)
Primary facility				1.26 (0.62–2.55)
**Usual place of care**				
Yes	Ref	Ref	NA	Ref
No	1.60 (1.08–2.37)*	1.62 (1.09–2.40)*		1.69 (1.13–2.53)*

^*^Denotes statistically significant at *p* < 0.005

Concerning the dignity domain, respondents in the poor socioeconomic class (OR = 1.67; 95% CI: 1.10–2.54), those with no formal education class (OR = 2.13; 95% CI: 1.08–4.21), those who used government-owned health facilities (OR = 1.75; 95% CI: 1.23–2.49) and those not using their usual health facility (OR = 1.60; 95% CI: 1.08–2.37) had significantly higher odds of experiencing poor dignity. For prompt attention domain, those in the poor WI (OR = 1.51; 95% CI: 1.01–2.25), users of government-owned facilities (OR = 1.46; 95% CI: 1.06–2.02) and those using facilities other than their usual place of care (OR = 1.62; 95% CI: 1.09–2.40) had higher odds of experiencing poor prompt attention. While significantly higher odds of poor amenities were observed by those in poor socioeconomic groups (OR = 1.53; 95% CI: 1.02– 2.31), users of government-owned facilities (OR = 2.16; 95% CI: 1.54–3.04) and rural respondents (OR = 1.84; 95% CI: 1.35–2.52). For total HSR, those who had no formal education (OR = 2.81; 95% CI: 1.35–5.86), primary education (OR = 2.19; 95% CI: 1.28–3.75), poor socioeconomic class (OR = 1.86; 95% CI: 1.23–2.80), users of government-owned facilities (OR = 1.56; 95% CI: 1.11–2.19) and those not using their usual facility (OR = 1.69; 95% CI: 1.13–2.53) had significantly higher odds of poor overall HSR.

## Discussion

This study was conducted to assess the level & distribution of HSR and identify the domains of HSR that are most important to people in Oyo State, Southwest Nigeria. In addition, the factors associated with experiencing poor HSR were also determined.

### Level of Health System responsiveness

The overall level of HSR of about 47.0% found in this study is similar to findings in Ghana 47.1% but lower than 62% for India, 66.9% in South Africa when vignettes and non-parametric analysis methods were used to correct patient reporting heterogeneity as was done in this study [47]. It is also similar to the 43.2% among insured clients in Egypt [58], 46.4% among HIV/AIDS clients in Ethiopia [59], but lower than the 53% among mental health clients in Iran, [60] 53% among pregnant women in Ethiopia [61], 59% in a hospital-based study in Enugu Nigeria [38], 67% among primary health care users in Bangladesh [62] and 69.1% among primary health care in Tanzania [63]. Although, the variations in the latter group of studies could have been due to methodological differences, notwithstanding the results suggest the level of HSR in the state is low and requires some reforms.

The level of HSR varied across the domains. Overall, the finding of confidentiality, dignity and choice being highly rated, and prompt attention as the least rated is consistent with findings in South Africa [57], Tanzania [63], Ethiopia [61] and Brazil [64]. It is also partly similar to a study carried out in Kaduna, Nigeria where quality of amenities, dignity and choice were the best performing domains and prompt attention was almost the least rated [40]. In contrast, a study done in Enugu found that choice was one of the worst-performing domains, while prompt attention was one of the best performing domains [38]. A plausible explanation for the ratings gotten in this study is that a good proportion of respondents trust health care workers (HCW) to be confidential with the information provided and have no reason to doubt the confidentiality of the information provided. Furthermore, prompt attention is seen as very important by most respondents, therefore respondents might have been more critical in assessing the domain leading to the low ratings by the respondents. One reason why choice domain might have been rated highly is that the domain is operationalized in terms of both opportunity to choose a provider and to continue with the same provider if one wants to [6]. The high score for dignity and low score for communication is intriguing and could suggest that while health workers are willing to respect the dignity of users, they are not able to communicate effectively due to lack of time.

### Distribution of Health System responsiveness

The WHO framework focuses not only on the average levels of HSR but also on the distribution (inequalities). Inequalities are assessed by considering the distribution of responsiveness scores across individuals. As such, improvements in HSR must not only focus on average levels of HSR but also on achieving equity and fairness. It is pertinent to note that comparisons with other studies are limited due to the differences in the measurements of inequalities in HSR. While some studies use concentration index or the Gini coefficient [29], others use measures such as the Abul Naga–Yalcin index [30], notwithstanding, this study contributes to the growing knowledge on inequalities in HSR. The overall inequality index in this study is closer to 0 than to 1, this suggests that inequalities in HSR in the state are quite low. Nonetheless, there are some disparities observed in the way individuals were treated by the health system. A small percentage of respondents, about 3% said they experienced discrimination by the health system in the state, this is less than the 11.9% in South Africa [18] and 25% in Bangladesh [62]. The inequalities in HSR where they exist must be addressed as they can worsen the existing barriers in access to health care services and may contribute to poor health outcomes in the long run.

In this study, prompt attention, choice and communication showed the most inequalities, as such should be targeted for reforms. This is findings are in line with studies done in Wuhan and Europe that also found that the domain of prompt attention had the most inequalities [29, 30]. However it is partly in contrast to a study in 16 OECD countries that found that autonomy domain was the most unequally distributed, although the inequality in prompt attention was also high [51]. The finding here could be due to the fact that the domain of prompt attention included the time needed to get to the hospital and this could be influenced by socioeconomic status. Those in poor socioeconomic class can have difficulties in geographical access to health facilities, especially in low-income countries like Nigeria [65]. The findings imply that policies should be formulated to reduce these inequalities by ensuring groups like the uneducated, low socioeconomic class, who experienced low levels of HSR enjoy improved responsiveness.

### Most important domains of Health System responsiveness

For the importance of the domains of HSR, the finding of communication, prompt attention, dignity and quality of basic amenities being rated as the most important domains is similar to studies done in Lagos [39] and Kaduna, Nigeria [40] and elsewhere [58, 66]. It is also partly similar to the study in Enugu, Nigeria [38] and the multi-country survey study done by the WHO [67] in 41 countries including Nigeria that found prompt attention as the most important domain followed by dignity and communication. A plausible explanation for the findings is that HCWs in Nigeria are few, usually overworked and do not take time to explain symptoms and illness to patients and don’t wait for patients’ feedback [68, 69] as evidenced by the poor rating of the domain in this study. Additionally, prompt attention is usually important in the setting of outpatient care that was employed for this study.

Choice was rated as the least important out of seven domains and is in keeping with studies done in Lagos, Nigeria [39], Africa [58] and elsewhere [50, 66]. This can be explained by the fact that most patients generally tend to trust HCW and don’t mind which doctor or nurse they see at the health facilities. These findings imply that health systems and facility managers need to focus on the important domains of HSR for improvements. It is pertinent to note that while we improve some domains of HSR, we do not compromise the levels of other domains. Active monitoring of health services might help promote and enhance these domains for users of the health system.

### Factors Associated with Health System responsiveness

This study found statistically significant determinants for the different domains of HSR after controlling for confounders using the logistic regression analysis. The association between poor socioeconomic class and poor autonomy is consistent with findings in India [27]. This is attributed in most parts to the fact that empowerment and financial autonomy are linked to the ability to make informed choices and these individuals may refuse medical treatment where need be [70, 71]. The finding of those having no formal education or primary education as the highest level of education being associated with poor communication is similar to findings across Europe [56]. This can be explained by the reason that poorly educated individuals might have difficulties communicating with health care workers. For choice domain, those in poor socioeconomic groups experiencing fewer options in the choice of care providers is similar to findings in India [27]. An explanation for this is that those in low socioeconomic groups have less purchasing power and as such are not able to have several options when it pertains to healthcare. Furthermore, the finding of rural respondents experiencing poor choice is similar to findings in China and India [27, 49].This might be because urban areas have more health facilities and more health workers than rural areas. Also, the finding of users of government-owned facilities experiencing poorer choice is similar to a study done in Nigeria and Iran [40, 72].

For the dignity domain, this study’s finding of low socioeconomic groups and the least educated having higher odds of experiencing poor dignity is similar to findings in Nigeria, India and Bangladesh [27, 40, 62]. This can be explained by the fact that a higher socioeconomic class can increase the chance of being treated with respect. In this study users of public facilities were significantly more likely to experience poor dignity which is consistent with other studies done in Nigeria [39, 40]. This is partly explained by the reason that physical examination in government facilities might be done in a way that does not respect individual privacy. In the prompt attention domain, the finding of those in low socioeconomic groups experiencing poor prompt attention is similar to studies done in Nigeria [38] but different from a study in China [49] that found poor socioeconomic groups have better prompt attention. The finding in this study might be because those in lower socioeconomic groups are more likely to use government facilities as these are usually cheaper, but typically have long waiting times as found in this study. Users of government-owned facilities, in turn, were significantly more likely to experience poor prompt attention which is consistent with other studies done in Nigeria [39, 40]. This may be explained by the fact that government-owned health facilities are often overcrowded leading to long waiting times. More so, appointment times in private-owned hospitals are more staggered. In addition, the finding of respondents not using their usual place of care experiencing poor prompt attention is novel to our knowledge but not surprising. This can be because clients not using their usual place of care might have difficulties navigating new facilities and are interacting with health workers for the first time causing them to wait longer.

For quality of basic amenities, respondents in poor socioeconomic groups were significantly more likely to experience poor quality of amenities which is similar to findings in Kaduna, Nigeria [40]. Also, rural respondents were significantly more likely to experience poor quality of amenities as shown in this study and a study in India [27]suggesting that urban facilities had a better quality of amenities. Similarly, users of government-owned hospitals had significantly higher odds of experiencing poor amenities as found by another study in Lagos, Nigeria [39]. Some reasons for these findings could be because those in high socioeconomic class can afford facilities with better amenities, urban facilities are better funded than their rural counterparts, and private facilities are run as a business and are competitive, therefore typically having better amenities than government-owned facilities. An improvement in amenities might go a long way in getting individuals to use government-owned health facilities.

For total HSR, the finding of respondents with low educational levels experiencing poor HSR is similar to other studies done in similar climes [40, 52, 56, 73]. In contrast, some other studies found no association between education and poor HSR [47, 55, 57]. This may reflect the fact that higher education is likely to improve understanding and communication as well as being treated with respect [74]. This study’s finding of respondents not using their usual place of care as a determinant of experiencing poor HSR is new to our knowledge and can be attributed to the fact that when patients have the option, they will go back to the facilities they are comfortable with and where they enjoy improved responsiveness [75, 76]. In addition, perception of quality of care will influence the utilization of such facilities [77]. This also explains the reason why choice domain is measured in terms of the opportunity to choose a provider and to continue with the same provider if one wants to do so.

This study’s finding of government-owned facilities as a determinant of poor HSR is consistent with other studies in Africa [18, 39, 40, 54, 55, 57, 78]. The poor performance of government-owned providers can be attributed to a reluctance to stagger appointments, high patient numbers exceeding the capacity of the facilities to cope, poor quality of public health facilities and poor attitude of some HCW [79, 80]. Another plausible explanation is that private hospitals run a business model and are therefore likely to treat their patients with dignity, have cleaner facilities and promptly attend to them [79, 80]. Consequently, there should be a focus to improve HSR in government hospitals. This might lead to increased utilization where necessary and improved quality of care by all groups.

This study did not find significant associations between sociodemographic characteristics like age, gender & marital status and the domains of HSR as was found in other studies [38–40, 53]. Similarly, respondents with poor self-reported health status did not report any significant negative experience in all the domains of HSR that were found in other studies in Europe [56, 81, 82]. The differences in the results might have been due to the approaches used in the way data was collected and analyzed. Contextual factors like culture might have also played a role in the findings.

## Strengths and limitations of the study

The strengths of this study: To begin with, there was a very good response rate with 94.3% of those who met the inclusion criteria completing their interviews. In addition, vignettes along with statistical analysis were used to assess HSR. This eliminated response bias that is due to respondents’ expectations, thereby ensuring that the results are more precise and comparable. Furthermore, this study focused on the evaluation of the entire state health system as against just evaluation of small sections or services of the health system. Finally, respondents were interviewed in their homes, as such eliminates the response bias that occurred in previous studies carried out in this clime that were hospital-based [38, 39, 83]. Previous studies have also shown that respondents who answer surveys at home are more critical compared with respondents who are interviewed in the hospital [84].

Some of the limitations of the study are as follows: firstly, HSR was assessed for users of the formal health system, and opinions of non-users or users of the informal health system were not taken into consideration. The implication is that the opinions of these groups of people are not accounted for. More so, it could not be established if responsiveness barriers were the reasons why these groups of people did not utilize the formal health system. This presents a gap that can be looked at in future studies. Secondly, the study was a community-based study where the respondents were asked questions in their homes, therefore health facility-based determinants of HSR like the health facility budget, personnel, services, and finance could not be ascertained or linked to respondents’ ratings of HSR. Also, community determinants of HSR like; health expenditure per capita can be difficult to ascertain, hence were excluded from this study. In addition, this study was a cross-sectional study as such causal relationships between exploratory variables and outcome (HSR) cannot be established. Finally, a limitation of the WHO methodology on HSR that was used in this study is that it measures just one encounter (the last encounter) with the health system rather than encounters over time. Despite these limitations, the results present a baseline metric for HSR in the state and country at large.

## Conclusion and recommendations

The overall level of HSR in Oyo State, Southwest Nigeria was generally low. Respondents experienced the lowest levels in the domains of prompt attention, communication and autonomy. The distribution of HSR showed some disparities with the domains of prompt attention, choice and communication showing the highest levels of inequalities. Communication, prompt attention and dignity were identified as the most important domains, while choice was rated as the least important.

The significant determinants of poor HSR (total) were having a low level of education, poor socioeconomic class, using a government-owned facility and not using the usual healthcare facility. Consequently, the low levels of HSR found, suggest that government and policymakers through their ministry of health and health care managers should embark on health system reforms that will help ensure that the levels of HSR in the state are improved upon, focusing on domains that performed poorly and those that were seen as important. This will lead to an overall improvement in the state and national health system.

## Supplementary Information

**Additional file 1: Appendix 1.** Questionnaire for HSR. **Appendix 2.**Adjusted (with vignettes ) Ambulatory scores of HSR . **Appendix 3.** Unadjusted Ambulatory scores of HSR.

## Data Availability

The datasets generated during and/or analysed during the current study are available from the corresponding author on reasonable request.

## Abbreviations

CI: Confidence Interval
EA: Enumeration Area
HCW: Health Care Workers
HIV: Human Immunodeficiency Virus
HSS: Health System Strengthening
HSR: Health System Responsiveness
IMD: Individual-mean Difference
IRB: Institutional Review Board
LGA: Local Government Area
NCD: Non-Communicable Diseases
NGO: Non-Governmental Organizations
OECD: Organization for Economic Co-operation and Development
OR: Odds Ratio
PC: Principal Component
PCA: Principal Component Analysis
RA: Research Assistant
SPSS: Statistical Package for Social Sciences
WHO: World Health Organization
WHR: World Health Report
WHS: World Health Survey
WI: Wealth Index
