# Supplementary material for: Assessment of the level and distribution of health system responsiveness in Oyo State, Nigeria
Source: BMC Health Serv Res. 2022 Jul 12;22:905. doi: 10.1186/s12913-022-08276-9 (PMC9281151; doi:10.1186/s12913-022-08276-9)
Supplement: Supplementary file 1 — Additional file 1: Appendix 1. Questionnaire for HSR. Appendix 2.Adjusted (with vignettes ) Ambulatory scores of HSR . Appendix 3. Unadjusted Ambulatory scores of HSR. [file 12913_2022_8276_MOESM1_ESM.docx]

**Online supplementary document**

**Adelabu et al. Assessment of the Level and Distribution of Health System Responsiveness in Oyo State, Nigeria**

**BMC Health services research**

Appendix 1. Questionnaire for HSR

Appendix 2. Ambulatory scores for the domains of HSR after adjusting with vignettes

Appendix 3. Unadjusted Ambulatory scores of HSR

#

# APPENDIX 1: QUESTIONNAIRE FOR USERS OF HEALTH SYSTEM

A Comparative Assessment of the Levels and Distribution of Health System Responsiveness in Urban and Rural Areas in Oyo State

**Identification**

LGA:……………………………………………………………………..

Ward (number and name):……………………………………………………

Location of LGA 1. Rural 2. Urban

**Section A: Socio-demographic**

**Instruction for interviewer: *Fill the responses into the column provided using the codes provided***

| **S/N** | **Information** | **Response** |
| --- | --- | --- |
| 1 | Age at last birthday (in years) |  |
| 2 | Record Gender as observed 1 Male 2 Female |  |
| 3 | What is your current marital status 1. Single, never married  2. Cohabiting 3. Married 4. Separated 5. Divorced 6. Widowed |  |
| 4 | What is your highest level of education completed 1. No formal 2. Primary 3. Secondary 4. Tertiary 5. Other (specify) |  |
| 5 | Religion 1. Christianity 2. Islam 3. Traditional 4. Other (specify) |  |
| 6 | Ethnicity/ Tribe 1. Yoruba 2. Hausa 3. Igbo 4. Other (specify) |  |
| 7 | Current occupation 1. Professional/Managerial 2. Skilled workers 3. Partially skilled. 4. Unskilled workers 5. Unemployed |  |
| 8 | What is your average monthly income (Naira) |  |
| 9 | What type of fuel do you use for cooking? Pick all that apply 1. Electricity 2. Natural gas 3. Kerosene 4. Coal 5. Charcoal 6. Wood 7. Animal dung 8. Agricultural shrubs/straw/ shrubs/ grass 9. No food cooked in household |  |
| 10 | What is the main source of drinking water in your household? 1. Piped water 2. Well 3. Borehole 4. Rainwater 5. Surface water (river/dam/lake/pond/stream) 6. Other (specify) |  |
| 11 | What kind of toilet facilities do you use in your household? 1. Flush toilet 2. Pit latrine 3. Bucket latrine 4. No facility/Bush/field 5. Other (specify) |  |
| 12 | What method of waste disposal do you use in your household? 1. Burning 2. Dumping 3. Burying 4. Government services 5. Other (specify) |  |
| 13 | How many rooms in total are in your household? (including rooms for sleeping and all other rooms) |  |
| 14 | How many people live in your household? |  |
| 15 | Does your household have the following items which are in **good working** condition? 1. Yes 2. No |  |
|  | 1. Electricity |  |
|  | 1. Radio |  |
|  | 1. Television |  |
|  | 1. Mobile telephone |  |
|  | 1. Refrigerator |  |
|  | 1. Air conditioning |  |
|  | 1. A generating set |  |
|  | 1. A computer |  |
|  | 1. Motor cycle |  |
|  | 1. Car or truck |  |
| 16 | Do you own any of the assets listed? 1. Yes 2. No |  |
|  | 1. Land |  |
|  | 1. Livestock |  |
|  | 1. House |  |
|  | 1. Gold/ jewellery |  |
|  | 1. Shares/ Investments |  |
|  | VI Other (specify) |  |

**Section B: Self-reported health description**

| S/N | Question | Options | Response | Skip to |
| --- | --- | --- | --- | --- |
| 17 | In general, how would you rate your health today? | Excellent............................**1**  Very good..........................**2** Good…..............................**3**  Fair.....................................**4**  Poor…............................... **5** |  |  |
| 18 | Do you have any disability | Yes…………1  No ………….2 |  |  |
| 19 | Do you have any chronic diseases? E.g diabetes, hypertension | Yes ………..1  No…………2 |  |  |

**Section C: Needing Health Care and General Evaluation of Health facility**

***Instruction for Interviewer:* The questions asked in this section are focused on the last visit to the health facility**.

| S/N | Question | Options | Response | Skip to |
| --- | --- | --- | --- | --- |
| 20 | Have you [your child] received any health care in the last 12 months? | Yes............................... **1**  No.................................2 |  |  |
| 21 | When was your [your child] last visit to a health facility or provider? | Was it…  In the last 30 days?.......**1**  In the last 3 months?.....**2**  In the last 6 months.......**3**  Between 6 and 12 months.....**4**  Don’t remember.............**5** |  |  |
| 22 | Was the last need for health care for yourself or for your child? | Yourself ……..1  Your child …………2 |  | If no, skip to 25 |
| 23 | What is the sex of the child? | Female ……….1  Male…………...2 |  |  |
| 24 | What is the date of birth of the child? | DD/MM/YY |  |  |
| 25 | What was the reason for your last health facility visit? | Acutely ill……………1  Chronic condition……..2  Preventive care; immunization…3  Others…….. |  |  |
| 26 | What was the name of the health care facility? | **(Please fill in name of facility,**  **e.g. Adeoye Clinic)** |  |  |
| 27 | What type of facility did you [your child] receive care? | Government hospital….1  Private hospital/clinic…2  NGO…………………..3  Others…………………4 |  |  |
| 28 | Level of care of facility attended | Primary…….1  Secondary….2  Tertiary…….3 |  |  |
| 29 | Was [name provided in 26] your usual place of care? | Yes............................... **1**  No.................................2 |  |  |
| 30 | Which was the last health care provider you visited? | **1.** Medical doctor (including gynecologist, psychiatrist, ophthalmologist, etc.)  **2.** Nurse  **3.** Midwife  **4.** Dentist  **5.** Physiotherapist or chiropractor  **6**. others |  |  |
| 31 | Thinking about your [child's] last visit, how much did you or your household pay for your care (₦): [Interviewer: only write 0 if the service was free. If a person did not have tests or drugs put X for “Not applicable, not have” and 99 if person does not know] |  |  |  |
|  | [Health care provider's] fees |  |  |  |
|  | Medicines |  |  |  |
|  | Tests |  |  |  |
|  | Transport |  |  |  |
|  | Other |  |  |  |
| 32 | Are you covered by any public or private health insurance funds? | Yes = 1  NO = 2 |  |  |
| 33 | In the last 12 months did you feel that you were treated worse by health care providers for any of the following reasons? Because of your | Yes = 1  NO = 2 |  |  |
|  | 1. Age |  |  |  |
|  | 1. Sex |  |  |  |
|  | 1. Tribe |  |  |  |
|  | 1. Lack of money |  |  |  |
|  | 1. Social class |  |  |  |
|  | 1. Type of illness |  |  |  |
|  | 1. Nationality |  |  |  |

**SECTION D: Health System Responsiveness**

| S/N | **Question** | Options | Response | Skip to |
| --- | --- | --- | --- | --- |
|  | **Prompt attention** | The next questions are about  how promptly you got care |  |  |
| 34. | Thinking about your [child's] last visit, how long did it take you to get to the health facility? (minutes) | …………minutes  ………… hour |  |  |
| 35. | For your [child's] last visit, how would you rate the travelling time to [the health care provider]? | Very good............................**1**  Good....................................**2**  Moderate.............................**3**  Bad......................................**4**  Very bad………………….5 |  |  |
| 36. | Thinking about your [child's] last visit, how long did you have to wait from the time that you wanted care to the time that you received care? | …………minutes  ………… hour  ………….days  ………… weeks  ………… months |  |  |
| 37. | For your [child's] last visit, how would you rate the amount of time you waited before being attended to? | Very good............................**1**  Good....................................**2**  Moderate.............................**3**  Bad......................................**4**  Very bad………………….5 |  |  |
| 38. | In the last 12 months have you needed any laboratory tests or examinations?  Some examples of tests or special examinations are blood tests, scans or X-rays. | Yes............................... **1**  No.................................2 |  | If No, Skip to 41 |
| 39 | Generally, how long did you have to wait before you could get the laboratory tests or examinations done? | Got them same day...............**1**  1-2 days................................**2**  3-5 days................................**3**  6-10 days..............................**4**  More than 10 days (specify).**5** |  |  |
| 40 | For your [child's] last visit, how would you rate the amount of time you waited before getting you laboratory or examinations done? | Very good............................**1**  Good....................................**2**  Moderate.............................**3**  Bad......................................**4**  Very bad………………….**5** |  |  |
|  | **Dignity** | The next questions are about the dignity with which you were treated when you sought health care |  |  |
| 41 | For your [child's] last visit, how would you rate your experience of being greeted and talked to respectfully? | Very good............................**1**  Good....................................**2**  Moderate.............................**3**  Bad......................................**4**  Very bad………………..…**5** |  |  |
| 42 | For your [child's] last visit, how would you rate the way your privacy was respected during physical examinations and treatments? | Very good............................**1**  Good....................................**2**  Moderate.............................**3**  Bad......................................**4**  Very bad………………….**5** |  |  |
|  | **Communication** | The next questions are about how healthcare providers communicated with you when you sought healthcare |  |  |
| 43 | For your [child's] last visit, how would you rate the experience of how clearly health care providers explained things to you? | Very good..............................**1**  Good.......................................**2**  Moderate................................**3**  Bad………………….............4  Very bad…………………….5 |  |  |
| 44 | For your [child's] last visit, how would you rate the experience of how clearly health care providers listened carefully to you? | Very good..............................**1**  Good.......................................**2**  Moderate................................**3**  Bad………………….............4  Very bad…………………….5 |  |  |
| 45 | For your [child's] last visit, how would you rate your experience of getting enough time to ask questions about your health problem or treatment? | Very good..............................**1**  Good.......................................**2**  Moderate................................**3**  Bad………………….............4  Very bad…………………….5 |  |  |
|  | **Autonomy** | As part of your care, decisions are made about which treatments or tests to give. The next questions are your involvement in decisions about the care and treatment you received in your [ child’s] last visit |  |  |
| 46 | For your [child's] last visit, how would you rate your experience of getting information about other types of treatments or tests? | Very good..............................**1**  Good......................................**2**  Moderate...............................**3**  Bad………………….............4  Very bad…………………….5 |  |  |
| 47 | For your [child's] last visit, how would you rate your experience of being involved in making decisions about your health care or treatment? | Very good..............................**1**  Good......................................**2**  Moderate...............................**3**  Bad………………….............4  Very bad…………………….5 |  |  |
|  | **Confidentiality of Information** | The next questions are about your experience of confidentiality of information in the health services. |  |  |
| 48 | For your [child's] last visit, how would you rate the way the health services ensured you could talk privately to health care providers? | Very good..............................**1**  Good.......................................**2**  Moderate................................**3**  Bad………………….............4  Very bad…………………….5 |  |  |
| 49 | For your [child's] last visit, how would you rate the way your personal information was kept confidential? | Very good..............................**1**  Good.......................................**2**  Moderate................................**3**  Bad………………….............4  Very bad…………………….5 |  |  |
|  | **Choice** | The next question are about the choice of health care providers you have. |  |  |
| 50 | For your [child's] last visit, how would you rate the freedom you had to choose your [health care provider or service]? | Very good............................**1**  Good....................................**2**  Moderate.............................**3**  Bad......................................**4**  Very bad..............................**5** |  |  |
| 51 | For your [child's] last visit, how would you rate the freedom you had at continuity of care by one health care provider? | Very good............................**1**  Good....................................**2**  Moderate.............................**3**  Bad......................................**4**  Very bad..............................**5** |  |  |
|  | **Quality of Surroundings or Environment** | The next questions are about the environment or the surroundings at the places you go to for health care. |  |  |
| 52 | For your [child's] last visit, how would you rate the cleanliness of the rooms inside the facility, including toilets? | Very good............................**1**  Good....................................**2**  Moderate.............................**3**  Bad......................................**4**  Very bad..............................**5** |  |  |
| 53 | For your [child's] last visit, how would you rate the amount of space in the waiting and examination rooms? | Very good............................**1**  Good....................................**2**  Moderate.............................**3**  Bad......................................**4**  Very bad..............................**5** |  |  |

SECTION E: Anchoring vignette for health system Responsiveness

|  | READ OUT | Very Good | Good | Moderate | Bad | Very  Bad | Don’t know |
| --- | --- | --- | --- | --- | --- | --- | --- |
|  | Autonomy Domain |  |  |  |  |  |  |
| 54 | When [Sarah] wanted treatment for her swollen limbs, the nurses at her clinic discussed many possible treatments with her. They discussed all the pro's and con's of each treatment with her and then recommended one to her. How would you rate [Sarah's] experience of being involved in making decisions about her health care or treatment? |  |  |  |  |  |  |
| 55 | When [Grace] had treatment for infertility, the doctor gave her some pills and asked her to return in two weeks. He didn't ask her whether she wanted to know anything about her health condition nor suggest different alternatives that might have suited her better. How would you rate [Grace] experience of being involved in making decisions about her health care or treatment? |  |  |  |  |  |  |
|  | Communication Domain |  |  |  |  |  |  |
| 56 | [Yetunde] went to the emergency clinic with stomach pain. The doctor explained to [Yetunde] her condition and the treatment. [Yetunde] asked him some questions and the doctor explained things using examples that were familiar to her until she understood everything. How would you rate her experience of how clearly health care providers explained things to her? |  |  |  |  |  |  |
| 57 | [Rose] cannot write or read. She went to the doctor because she was feeling dizzy. The doctor didn't have time to answer her questions or to explain anything. He sent her away with a piece of paper without telling her what it said. How would you rate her experience of how clearly health care providers explained things to her? |  |  |  |  |  |  |
|  | Confidentiality Domain |  |  |  |  |  |  |
| 58 | [Simon] was speaking to his doctor about an embarrassing problem. There was a friend and a neighbor of his in the crowded waiting room and because of the noise the doctor had to shout when telling [Simon] the treatment he needed. How would you rate the way the health services ensured [Simon] could talk privately to health care providers? |  |  |  |  |  |  |
| 59 | [Kehinde] visited the doctor regularly. His doctor always took [Kehinde] to a private room before discussing his illness. The doctor was aware that [Kehinde] was very sensitive about his health condition and would never talk about it to anyone or in front of anyone without Kehinde’s permission. How would you rate the way the health services ensured [Kehinde] could talk privately to health care providers? |  |  |  |  |  |  |
|  | Choice Domain |  |  |  |  |  |  |
| 60 | When [Lola] had fertility problems, she had to travel from her small village to the closest town where they only had one male doctor. [Lola] wanted to see a female doctor but she had no choice. How would you rate [Lola's] freedom to choose her health care provider? |  |  |  |  |  |  |
| 61 | [Ken] lives in a town where there are lots of doctors and clinics. He tried one doctor but he didn't like him so he changed doctors. This was easy to do because he could go where he wanted. How would you rate [Ken's] freedom to choose his health care provider? |  |  |  |  |  |  |
|  | Dignity Domain |  |  |  |  |  |  |
| 62 | [Muyiwa] had a bad flu. He went to the clinic. The nurse expressed concern about [Muyiwa]'s cough and called the doctor, who gave [Muyiwa] a full chest examination behind a large screen that hid him from the view of other patients. How would you rate his experience of being talked to and treated respectfully? |  |  |  |  |  |  |
| 63 | [Saheed] has Tuberculosis. When he goes to his health centre the nurses do not talk to him and deliberately ignore him. During examinations, his clothes are removed and he is made to wait, half-naked in the waiting room. How would you rate his experience of being talked to and treated respectfully? |  |  |  |  |  |  |
|  | Prompt attention Domain |  |  |  |  |  |  |
| 64 | [Segun] broke his leg. It took an hour to be driven to the nearest hospital. He was in pain but had to wait an hour for the surgeon and was only operated on the next day. How would you rate the amount of time he waited before being attended to? |  |  |  |  |  |  |
| 65 | [Akala]'s child became seriously sick. [Akala] called an ambulance that arrived after 10 minutes and within 5 minutes they were at the hospital and the doctors were treating the child. How would you rate the amount of time he waited before being attended to? |  |  |  |  |  |  |
|  | Quality of basic amenities Domain |  |  |  |  |  |  |
| 66 | [Kamal] has a nervous breakdown and had to spend 3 months in the past year in the local hospital. He had to sleep on an uncomfortable mattress with no sheets. There were 30 other patients in the same dormitory style ward and the toilets would smell, because they were not cleaned. He came back with a skin infection, because he couldn't wash regularly and there were insects in the bed. How would you rate the cleanliness of the rooms inside the facility, including toilets? |  |  |  |  |  |  |
| 67 | [Moses] had his own room in the hospital and shared a bathroom with two others. The room and bathroom were cleaned frequently and had fresh air. How would you rate the cleanliness of the rooms inside the facility, including toilets? |  |  |  |  |  |  |

SECTION F: IMPORTANCE OF RESPONSIVENESS DOMAINS

| S/N | Responsiveness Domains | Extreme Important | Very Important | Moderately Important | Slightly Important | Not Important |
| --- | --- | --- | --- | --- | --- | --- |
| 68 | How important is "**respectful treatment**" to you. This means being shown respect when greeted by and when talking to health care Providers having physical examinations conducted in a way that respects your cultural norms Would you say it is: |  |  |  |  |  |
| 69 | How important is "**confidentiality of personal information**" to you. This means having information about your health and other personal information kept confidential having conversations with health care providers without other people over hearing Would you say it is: |  |  |  |  |  |
| 70 | How important is "**convenient travel and short waiting times**" to you. This means having short travel times and convenient access to health care facilities having short waiting times for consultations and hospital admissions Would you say it is: |  |  |  |  |  |
| 71 | How important is "**choice of health care providers**" to you. This means, being able to choose your health care provider (place or person) being able to consult for a second opinion or with a specialist if so desired Would you say it is: |  |  |  |  |  |
| 72 | How important is "**involvement in decision making**" to you. This means being involved as much as you want in deciding about your health care freedom to discuss other treatment options or care regimes if you want. Would you say it is: |  |  |  |  |  |
| 73 | How important are "g**ood quality surroundings**" to you? This means having enough space, seating and fresh air in the waiting rooms, examination rooms and hospital ward shaving a clean facility (including clean toilets) Would you say it is: |  |  |  |  |  |
| 74 | How important is "**contact with the outside world**” to you? This mean shaving family and friends visit you as much as you want when you are a patient in hospital being able to keep in contact with family and friends and to have information about what is happening outside the hospital Would you say it is: |  |  |  |  |  |
| 75 | How important is "**clarity of communication**" to you. This means having the health care providers explain things in a way you can understand having enough time to ask questions if you don’t understand something. Would you say it is: |  |  |  |  |  |

**76. Ranking of importance of responsiveness domains**

|  | **RANK 1 (MOST important domain)** |  |
| --- | --- | --- |
|  | **RANK 2** |  |
|  | **RANK 3** |  |
|  | **RANK 4** |  |
|  | **RANK 8 (LEAST important domain)** |  |

**Thank you for your time.**

Appendix 2: Table showing HSR Scores after adjustment with vignettes

| **Domains of HSR** | **Total (N = 717)** |
| --- | --- |
|  | **Mean and SD** |
| **Autonomy** | $54.7\pm1.1$ |
| **Choice** | $59.7\pm1.2$ |
| **Communication** | $53.4\pm1.1$ |
| **Confidentiality of Information** | $68.3\pm1.1$ |
| **Dignity** | $62.7\pm1.2$ |
| **Prompt Attention** | $49.9\pm1.2$ |
| **Quality of Environment** | $58.5\pm1.1$ |
| **Overall Level of Responsiveness** | $58.2\pm0.6$ |

Appendix 3: Table showing HSR Scores before adjustment with vignettes

| **Domains of HSR** | **Total (N = 717)** |
| --- | --- |
|  | **Mean and SD** |
| **Autonomy** | $74.5\pm1.4$ |
| **Choice** | $66.7\pm2.2$ |
| **Communication** | $83.8\pm1.2$ |
| **Confidentiality of Information** | $84.3\pm1.9$ |
| **Dignity** | $84.8\pm1.4$ |
| **Prompt Attention** | $81.0\pm1.2$ |
| **Quality of Environment** | $86.2\pm1.3$ |
| **Overall Level of Responsiveness** | $80.2\pm0.8$ |
